# The common pathophysiologic threads between Asian Indian diabetic’s ‘Thin Fat Phenotype’ and partial lipodystrophy: the peripheral adipose tissue transcriptomic evidences

**DOI:** 10.1080/21623945.2020.1776082

**Published:** 2020-06-03

**Authors:** Aditya Saxena, Pradeep Tiwari, Nitin Wahi, Anshul Kumar, Sandeep Kumar Mathur

**Affiliations:** a Department of Biotechnology, Institute of Applied Sciences and Humanities, GLA University, Mathura, India; b Department of Endocrinology, Sawai Man Singh Medical College and Hospital, Jaipur, India; c Department of Biotechnology and Bioinformatics, Birla Institute of Scientific Research (BISR), Jaipur, India; d Department of Chemistry, School of Basic Sciences, Manipal University Jaipur, Jaipur, India; e Department of Bioinfoirmatics, Pathfinder Research and Training Foundation, Gr. Noida, India

**Keywords:** Lipodystrophy, type 2 diabetes, network biology, WGCNA

## Abstract

T2D is a complex disease with poorly understood mechanisms. In Asian Indians, it is associated with “thin fat” phenotype which resembles with partial lipodystrophy. We hypothesized that disturbed expression of lipodystrophy genes might play a role in T2D pathogenesis. Therefore, we attempted to establish a link between these two diseases by studying the overlap between the network of lipodystrophy genes and the differentially expressed genes (DEGs) in the peripheral subcutaneous adipose tissue of Asian Indians diabetics. We found that 16, out of 138 lipodystrophy genes were differentially regulated in diabetics and around 18% overlap between their network and the DEGs; the expression level of lipodystrophy genes showed an association with disease-related intermediate phenotypic traits among diabetics but not in the control group. We also attempted to individualize the diabetic patients based on ±2 fold altered expression of lipodystrophy genes as compared to their average expression in the control group. In conclusion, significant overlap exists between some of the lipodystrophy genes and their network with DEGs in the peripheral adipose tissue in diabetics. They possibly play a role in the pathogenesis of diabetes and individualization of diabetics is possible based on their altered expression in their peripheral adipose tissue.

## Introduction

South Asians, especially Indians have a phenotype which is characterized by an excessive accumulation of adipose tissue for a given BMI (*kg/m^2^*) and they have been variously termed as ‘metabolically obese’ or ‘thin fat’ individuals. Their body fat distribution is further characterized by excess central/visceral fat deposition and a lesser peripheral subcutaneous fat deposition [1]. Interestingly, the occurrence of T2D in them is linked with greater degrees of this peculiar fat distribution pattern [2]. T2D subjects as compared to BMI matched non-diabetics were found to have less peripheral and more intra-abdominal fat. This pattern of body fat distribution has also been reported in the patients suffering from a rare genetic syndrome called ‘partial lipodystrophy’ [3]. Additionally, both of these disorders not only share common body fat distribution patterns but the consequent metabolic pathophysiology in them is also along the same lines [4]. The key elements of this pathophysiology are: The failure to store excess fat in protective depots like subcutaneous or peripheral adipose tissue, abnormal adipo-cytokinemia, ectopic fat deposition in vital organs like the liver, pancreas, blood vessels, muscle, etc., high insulin resistance, dyslipidemia, and heightened diabetic and atherosclerotic risk.

Despite the phenotypic resemblance, the Asian Indians’ thin fat phenotype and lipodystrophy syndrome are genetically distinct diseases. Familial partial lipodystrophy is a monogenic disorder with Mendelian mode of transmission. The underlying genetic mutations and the consequent biochemical, cellular and molecular pathophysiology is well characterized in the majority of the pedigrees. Whereas, T2D is a complex multifactorial disease and its clinical picture is created by the interaction of several environmental and genetic factors such as frequent polymorphisms of multiple genes. These polymorphisms may be localized in the coding or regulatory regions of these genes and are present with different frequencies in an individual. Despite several genome-wide association studies (GWAS) done so far, its genetic basis is only partly understood. For example, in a genome-wide association study of T2D in Asian Indians, we found that the previously known and the newly identified loci on 2q21 together accounted for only 7.65% variance in the occurrence of diabetes in this population [5]. Moreover, how these genetic variants contribute to known aspects of diabetic pathophysiology still remains a mystery. In another study, we used a systems biology approach to unravel genome to phenome correlation in T2D [6]. Wherein, we examined the enrichment of pathways in genes identified in T2D GWAS and found that genes at lower significance threshold showed enrichment of insulin secretion related pathways. The physical and the genetic interaction network of these genes showed a robust enrichment of insulin signalling and other T2D pathophysiology related pathways including insulin secretion. In this study, we generated genome-wide gene expression profiles of multiple insulin-responsive tissues from diabetic and non-diabetic patients. Remarkably, the differentially expressed genes showed significant overlap with the network of GWAS shortlisted genes, with the intersection showing enrichment of several pathways consistent with T2D pathophysiology including the insulin signalling pathway. Therefore, these findings suggest that system biology approaches like the generation of physical and genetic interaction network of disease-linked genes and its overlap with transcription profiles in tissues could help in understanding the genome to phenome correlation in complex diseases.

We recently argued that the known aspects of the genetics of lipodystrophy syndromes could help in understanding the pathophysiology of diabetes and metabolic syndrome [7]. Our preliminary analysis suggests that the ‘thin fat’ phenotype of Asian Indian T2D subjects could be a functional genetic variant of the lipodystrophy syndrome. Some of the mutations in the coding sequence of the ‘Lipodystrophy Genes’ which have been well known to cause monogenic lipodystrophy syndrome were found differentially expressed in the subcutaneous adipose tissue of diabetics in this population. To further support this hypothesis, we applied system genomic strategies to examine the genetic and functional overlap between T2D and lipodystrophy syndrome. The specific objectives of this study were to investigate (1) Whether lipodystrophy genes are differentially expressed in the peripheral subcutaneous adipose tissue of diabetics, (2) Is there an overlap between these differentially Expressed Genes (DEGs) of T2D patients and the physical and genetic interaction network of lipodystrophy genes, (3) Pathophysiological significance of altered expression of lipodystrophy genes by assessing their correlation with measured intermediate traits in diabetics and controls and (4) Sub-clustering of diabetics on the basis of altered expression of any specific lipodystrophy genes in their peripheral adipose tissue.

## Material & method

Previously we have generated genome-wide transcription profiles in 60 adipose tissue biopsy samples (using Affymetrix primeview- gene chips) obtained from equal number of normal glucose tolerant (N = 30, *M: F* ratio 1:1) and type 2 diabetic (N = 30, *M: F* ratio 1:1) subjects in a cross-sectional study conducted at S.M.S. Medical College and hospital, Jaipur, India As gonadal hormones are known to influence adipogenesis, therefore sex could be a potential confounder in this study. The design of this study, where, male to female ratio was 1:1 in both the groups, all the subjects were above 50 years of age and the female subjects had attained menopause, would be expected to largely evade the influence of sex hormones in interpretation of results of this study.

The adipose tissue biopsies were obtained from femoral subcutaneous adipose tissues, therefore they represents genome-scale gene-expression profiles of peripheral adipose tissue in Asian Indians. This transcription profiling data is available at Gene Expression Omnibus database at NCBI with series number GSE78721. We also measured different intermediate traits for each subject such as *Cell size, HOMA-IR, HOMA-B, Hb1Ac, Triglycerides, Cholesterol, NEFA, VLDL, LDL, HDL, leptin, adiponectin, TNF-*α, *hsCRP, Serum creatinine*, and *Insulin*. Table 1 presents gender-wise measured anthropometric, and biochemical parameters. The clinical information with detailed methodology has been published elsewhere [8,9].

**Table 1.** Gender-wise measured anthropometric, and biochemical parameters in both NGT, and T2D groups (expressed as means± S.D.)

Gender	Female			Male		
Group	NGT	T2D	t test	NGT	T2D	t test
Age	65.80 ± 13.64	64.12 ± 8.15	0.65	61.95 ± 9.07	58.87 ± 9.17	0.33
Weight (kg)	52.10 ± 10.17	63.12 ± 16.06	0.02	55.80 ± 6.92	63.07 ± 11.02	0.04
BMI	21.49 ± 4.02	26.31 ± 6.96	0.02	20.28 ± 2.80	22.73 ± 3.25	0.03
W: H	0.97 ± 0.07	0.99 ± 0.10	0.50	1.03 ± 0.21	1.03 ± 0.07	0.90
Fasting Glucose (mg/dl)	90.42 ± 12.48	183.32 ± 72.45	0.00007	88.96 ± 9.69	204.36 ± 102.13	0.0006
Triglyceride	163.70 ± 79.26	154.65 ± 53.17	0.68	154.32 ± 44.02	144.57 ± 55.02	0.59
Total Cholesterol	195.60 ± 42.19	182.04 ± 35.58	0.30	180.65 ± 37.41	192.63 ± 66.90	0.56
HDL	40.90 ± 8.83	41.61 ± 4.00	0.75	40.47 ± 4.43	40.21 ± 7.62	0.91
LDL	87.39 ± 20.63	93.05 ± 21.91	0.43	94.28 ± 19.16	95.69 ± 33.11	0.89
VLDL	31.71 ± 8.44	51.09 ± 40.88	0.07	31.22 ± 6.79	33.92 ± 10.65	0.44
Serum Creatinine	1.04 ± 0.32	1.04 ± 0.38	1.00	0.99 ± 0.17	1.08 ± 0.20	0.22
HOMA-B	192.89 ± 252.9	115.68 ± 125.97	0.24	132.87 ± 87.65	126.59 ± 126.75	0.87
HOMA-R	2.45 ± 2.13	12.30 ± 11.38	0.002	1.83 ± 1.47	11.97 ± 7.16	0.00007
Insulin	11.16 ± 9.89	26.22 ± 19.00	0.01	8.25 ± 5.93	24.44 ± 10.77	0.00003
Hb1Ac (%)	5.45 ± 0.62	8.17 ± 1.38	0.0000002	5.32 ± 0.55	9.22 ± 2.75	0.00007
NEFA (mmol/L)	0.62 ± 0.30	0.63 ± 0.48	0.92	0.51 ± 0.26	0.66 ± 0.43	0.26
HsCRP (ng/ml)	7950.78 ± 5701.0	8769.12 ± 4779.9	0.64	7279.16 ± 5140.79	10,202.27 ± 4134.99	0.07
Leptin (pg/ml)	8150.77 ± 7945.67	19,144.99 ± 27,723.65	0.13	7810.80 ± 5691.17	8094.73 ± 6584.18	0.89
Adiponectin (ng/ml)	210.71 ± 116.19	236.74 ± 239.48	0.69	183.76 ± 117.78	208.99 ± 86.46	0.47
IL-6	33.33 ± 53.05	30.53 ± 33.71	0.85	22.02 ± 29.45	30.34 ± 46.60	0.55
TNF-α	25.42 ± 17.51	28.84 ± 35.04	0.72	32.70 ± 29.63	28.75 ± 35.79	0.73

In the present study, these transcription profiling datasets were furtherer processed using Bioconductor package *gcrma* [10]. A non-specific filtering step was then carried out to select high sensitivity probes using package *genefilter* [8]. Differential gene expression was estimated using *limma* [9]. *in silico* validation of DE genes with a known role in lipodystrophy (Lipo-DE) was done using CRowd Extracted Expression of Differential Signatures (CREEDS) database [11] which contains collection of gene expression signature extracted from NCBI-GEO database.

The Lipodystrophy genome was constructed from 138 protein-coding genes using DisGeNET database [12] which is a comprehensive platform integrating information on human disease-associated genes and variants in terms of evidences mined from Comparative Toxicogenomics Database (CTD), UniProt, from animal models (Rat Genome Database (RGD) and Mouse Genome Database (MGD)), inferred from Literature Human Gene Derived Network (LHGDN) and BeFree. Therefore, this list of 138 lipodystrophy-associated genes thus represents a comprehensive collection supported by all the available resources [13,14].

High confidence human protein-protein interaction network containing 15,134 proteins was obtained from HumanConsensusPathDB and StringDB using cytoscape app PhenomScape [15]. A sub-network of 3956 proteins containing 130 lipodystrophic proteins (~95% of 138 total proteins) and their first-degree neighbours was further constructed and dubbed as ‘Lipodystrophy – interactome’.

To identify the molecular association of lipodystrophy with type 2 diabetes, DEGs were mapped on lipodystrophy- interactome and a subnetwork containing, these DEGs and their first-degree neighbours were constructed. A further network reduction step was carried out by selecting only Lipodystrophy-DEGs and their first-degree neighbours.

This sub-network is expected to contain some key proteins participating in the molecular processes shared by both lipodystrophies and type 2 diabetes. However, since pleiotropic proteins likely give rise to multiple phenotypes that makes it difficult to interpret their functional meaning especially when the initial list of the genes (protein) is long. To address this problem, we carried out a network topology based prioritization of this sub-network using Cytoscape app – cytoHubba [15] which provides a user-friendly interface to explore important nodes in biological networks. We created two sub-networks containing the top 100 nodes with the highest 1) degree-centrality, 2) bottleneck-centrality and finally a union-network was constructed using network merge functionality of Cytoscape. This dual-metric approach for network-based prioritization of proteins thus provided us with a modest number of high confidence proteins for subsequent pathway-based functional analysis.

For pathway-based functional analysis of these selected genes, we used WebGestalt [16] – a long-standing, widely used web application for functional enrichment analysis against the human collection of the WikiPathway database [17].

Besides, carrying out network-based functional analysis of lipo-DE genes, we also attempted to ascertain whether genome-scale expression of lipodystrophy genes in diabetic individuals showed a statistically significant correlation with type 2 diabetes-associated intermediate traits (*Cell size, HOMA-IR, HOMA-B, Hb1Ac, Triglycerides, Cholesterol, NEFA, VLDL, LDL, HDL, leptin, adiponectin, TNF-*α, *hsCRP, Serum creatinine*, and *Insulin*) in comparison to normal glucose tolerant individuals using a system biology method, Weighted Gene Correlation Network Analysis (WGCNA) [18] which identifies modules of co-expressed genes and define these modules in separate colours as visual aid. It also estimates Module Eigengene (ME) as the first principal component of the expression matrix of the corresponding module and provides functionalities to relate these MEs with external traits.

WGCNA was carried out as follows -The normalized expression values of lipo-genes were matched with the corresponding intermediate traits and a single step network construction and module detection was used by selecting soft threshold power β = 15 to ensure scale-free topology of the network. MEs were then used to relate each modules with intermediate traits.

Lipodystrhophy genes (both DE and non-DE) with ±2 standard deviation expression difference with average mean expression of control group were then listed for each diabetic patient and their frequencies were estimated in the disease group.

## Results

**Figure 1.**
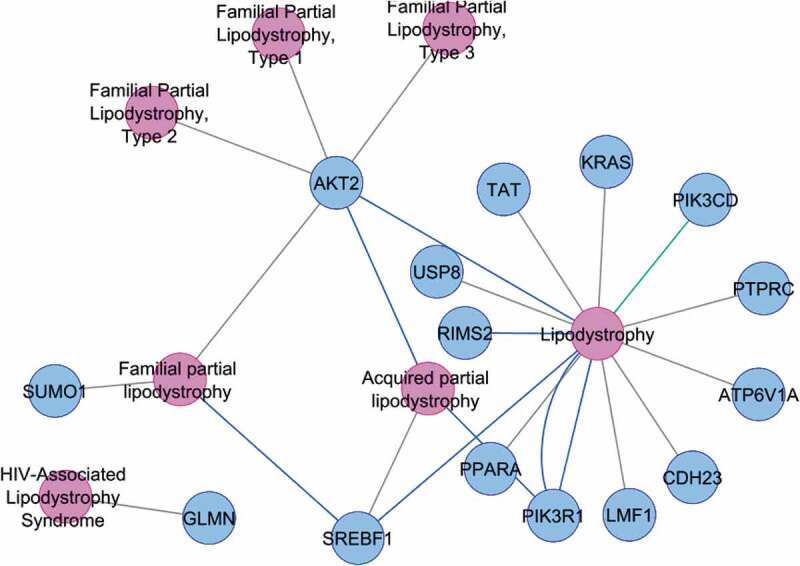
DE genes showing association with various sub-types of lipodystrophy

A total of 2537 genes were found to be Differentially expressed (DE) (*P < 0.05*) between normal and diabetic subjects; of them, seven up-regulated genes: *PTPRC, PIK3R1, ATP6V1A, IL7 R, KRAS, USP8*, and *GLMN* and nine down-regulated genes: *SUMO1, PPARA, CDH23, TAT, PIK3 CD, RIMS2, LMF1, AKT2*, and *SREBF1* have already been reported in lipodystrophy or its various subtypes using Cytoscape app – DisGeNET v5.0 analysis. (Figure 1)

To further validate the association of these lipodystrophy genes with diabetes, we searched the CREEDS database for studies involving single-gene perturbation, diseased versus normal tissue and drug perturbation. We found five normal vs. diabetic mice/rat gene expression studies (GSE2899, GSE2457, GSE11, GSE4616, and GSE2254; comprising a total of 74 expression datasets) showing the similar direction of expression for these 16 lipodystrophic genes.

In one microarray study – GSE2899, Lan *et al.*, (2005) compared gene expression profiles in adipose tissue, liver, muscle and islets between two mouse models of extreme leanness (lipodystrophy), and extreme obesity (*ob/ob*) and found that lipodystrophic-mice had a higher rate of hepatic lipogenesis. They hypothesized that there might be a shift in the ‘*lipogenic burden*’ from adipose tissue to other organs, such as the liver in animals with less lipogenic adipose tissues [19].

**Figure 2.**
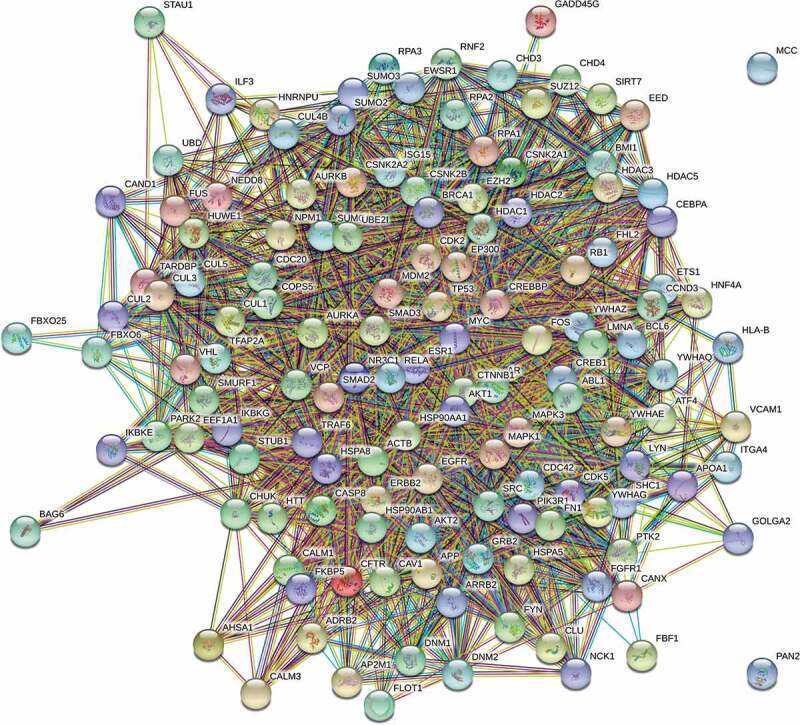
Interaction network of 135 lipodystrhophic-differentially expressed genes, prioritized by degree-centrality, and bottle-neck centrality

Network-based analysis to elucidate the shared T2D – Lipodystrophy aetiology was performed on lipodystrophy- interactome of 3956 proteins containing 111 lipodystrophy genes, 675 DEGs (~18% of 2537 DEGs) and their first-degree neighbours. Subsequent network reduction constructed a sub-network containing 3613 nodes including lipo-DE genes along with their first-degree neighbour and finally 135 proteins for downstream functional analysis (Figure 2) were selected from this sub-network based on network metric-based prioritization.

WebGstalt pathway analysis against human collection of WikiPathways enriched ten pathways (Table 2). For each enriched pathway, it calculates *expect value* (expected number of input genes that are annotated in the gene set), *Ratio* (Enrichment ratio, overlap/expect), *P-value* from hypergeometric test for Over Representation Analysis (ORA) and *FDR* for Corrected *P*-value for multiple testing for ORA. *P*-values and FDR were extremely significant; indeed top two pathways have zero chance of being false positive (*P* = 0; FDR = 0).

**Table 2.** Enriched pathways implicated by topologically prioritized genes

S. No.	Description	Size	Expect	Ratio	P Value	FDR
1	TGF-beta Signalling Pathway	132	3.1648	9.4794	0	0
2	Androgen receptor signalling pathway	90	2.1578	10.659	0	0
3	RANKL/RANK (Receptor activator of NFKB (ligand)) Signalling Pathway	55	1.3187	13.65	2.22E-16	3.23E-14
4	RAC1/PAK1/p38/MMP2 Pathway	68	1.6303	9.8139	2.57E-12	2.8E-10
5	Integrated Breast Cancer Pathway	151	3.6203	6.0768	5.03E-12	4.39E-10
6	Leptin signalling pathway	76	1.8221	8.7809	1.61E-11	1.17E-09
7	T-Cell antigen Receptor (TCR) Signalling Pathway	90	2.1578	7.8784	2.28E-11	1.42E-09
8	Breast cancer pathway	154	36,922	5.6876	5.89E-11	3.02E-09
9	IL-3 Signalling Pathway	49	1.1748	11.066	6.23E-11	3.02E-09
10	EGF/EGFR Signalling Pathway	162	3.884	5.4068	1.56E-10	6.82E-09

The role of *‘TGF-*β*signalling Pathway’* in causing lipodystrophy in Oral Submucous Fibrosis (OSMF) has been reported [20]. Transgenic mice, engineered for constitutive expression of TGF-β, have also been reported to develop lipodystrophy-like syndrome due to extreme fibrosis in the adipose depot [21]. Furthermore, Elevated TGF-β1 in humans has been shown to positively correlate with increased adiposity, a poor metabolic profile [22] and a higher risk for developing type 2 diabetes [23]. These findings point towards a common aetiology for lipodystrophy and Type 2 diabetes.

Activation of *‘Androgen receptor signalling pathway’* has also been reported to inhibit fat mass in cultured 3T3-L1 cells [24] and therefore may cause lipodystrophy like symptoms; the androgen receptor interacts with β-catenin protein in the Wnt pathway, thereby bypassing the canonical Wnt signalling – which functions as an adipogenic switch that represses adipogenesis when activated and initiates adipogenesis when it is turned off.

As NFκB signalling is the central pathway for inflammatory processes, enriched pathway, *‘RANKL/RANK (Receptor activator of NFKB (ligand) signalling Pathway’* points towards the role of inflammation in both the lipodystrophy and type 2 diabetes. However, a study has demonstrated that the infiltration of macrophages in lipodystrophic mice occurred in response to dead or dying adipocytes and did not induce insulin resistance as in the case of obesity [25].

Another enriched pathway ‘*Leptin signalling pathway*’ also links lipodystrophy with type 2 diabetes. Leptin is an adipokine and due to the loss of adipose tissue, lipodystrophies are generally associated with partial or total leptin deficiency that eventually causes neuroendocrine and metabolic abnormalities including insulin resistance and diabetes [26]. It is also interesting to note that leptin, even at concentrations as low as 0.1 ng/mL, induces significant tyrosine phosphorylation of the epidermal growth factor receptor (EGFR) [27]. Therefore, another enriched pathway ‘*EGF/EGFR signalling Pathway*’ is also significant in lipodystrophy; ErbB family members including EGF mediate the recruitment of new fat cells through proliferation, alternatively differentiation of preadipocytes and reduced expression of ErbB1 has been observed in insulin-resistant individuals [28]. It is, therefore, speculated that low leptin levels due to reduced adiposity may give rise to insulin resistance in lipodystrophies. Another pathway, ‘*T-Cell antigen Receptor (TCR) signalling Pathway*’ further points towards the role of immune dysregulation in both these diseases.

**Figure 3.**
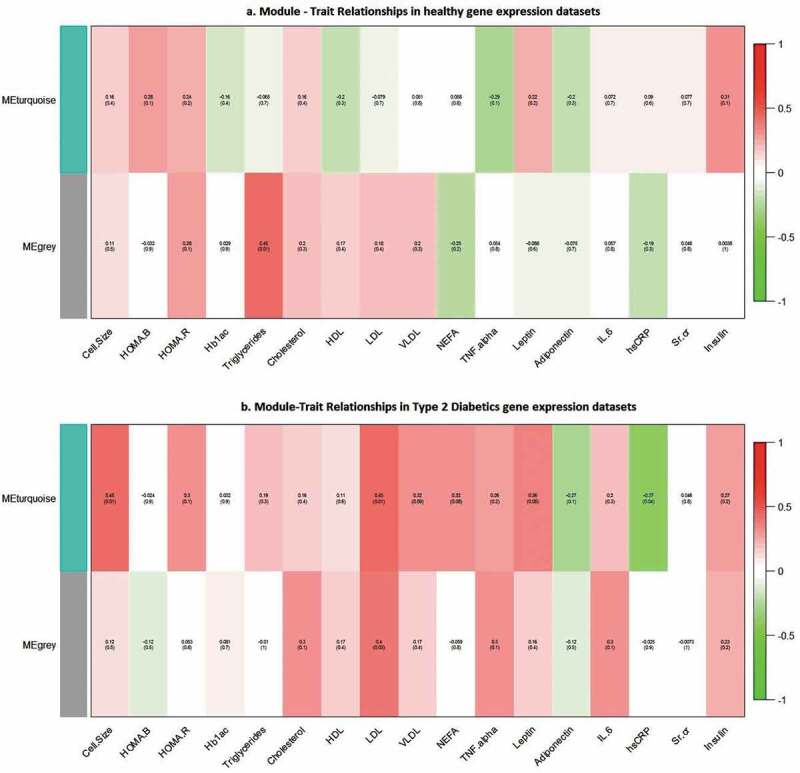
Results of WGCNA showing module – trait relationship between nondiabetic, and diabetic subjects. Correlations of traits with modules is shown by a colour-scale with green showing negative correlation and red, positive correlation

A total of 121 lipodystrophy genes were analysed at genomic-scale in diabetic and non-diabetic individuals separately using WGCNA and two modules (*grey*, and *turquoise*) were identified in both groups. WGCNA underscored our ‘common thread hypotheses’ between T2D and ipodystrophy, with diabetics showing correlation for more number of intermediate traits with lipo-genes. (Figure 3)

In nondiabetic individuals, only a single trait – Triglyceride showed positive correlation with ‘*grey*’ module (*P* < 0.01), whereas, in diabetic individuals, ‘*Turquoise*’ module showed a positive correlation with four traits – Cell size *(P *< 0.01), LDL (*P* < 0.01), Leptin (*P* < 0.05), and a negative correlation with hsCRP (*P* < 0.04); the ‘*grey*’ module showed positive correlation with only one module – LDL (*P* < 0.03).

Twenty five out of thirty diabetic individuals have been detected for atleast one lipodystrophic genes which shown ±2 fold up- and down regulation in expression from the average mean expression in the control group. (Table 3)

**Table 3.** List of up- and down-regulated lipodystrophic genes with ±2 fold expression differences from the average mean expression in the control group (see supplementary table S1 for full gene names)

S. No. /T2D individual	Up regulated	Down regulated
1	*B3GALT6, GHRL, HNF4A, TCN1*	*AGPAT2, APRT, CAVIN1, CCND3, CNBP, EMD, FABP4, GRN, HLA-DPB1, LMNA, LONP1, LPL, PDAP1, PPARA, TYROBP*
2	*ARTN, CCND3,CRABP2, EGF*	*HFE, PDAP1, PDE3B, POLD1, SLC28A2, SLC28A3*
3	*ATP6V0A2, ATP6V1A, CRABP2, ESR1*	*RARB*
4	*AGPAT1, ATP6V1A, CD68, LMF1*	*NR3C1, PARP2, PPARA, PSMB8, SLC28A1*
5	*CCL3, IL7 R, TNF*	*IL18*
6	*ATP6V1A, PPARA, SPRTN*	
7	*INSR, LEPR, PIK3R1, PPARA*	*USP8*
8	*RIMS2*	
9	*CCL3, FBN1, LIPE*	
10	*ADIPOR2, DIO2, INSR, LIPE*	*LMNB2, LPL, SERPINE1, TYMS,*
11	*CCL3, FBN1, LIPE*	
12	*FBN1, LEPR*	
13	*ADIPOR2, ATP6V0A2, B4GALT7, CAVIN1*	*CDH23, CIDEC, CXCL8, FAS, FBN1, IL7 R, LIPE, LMNB1, METTL9, PLIN1, PPOX, PTPRC, RETN, TCN1, TYMS*
14	*CDH23, LIPE, LMNB2*	
15	*CAVIN1, CDH23, CIDEC, FBN1*	*LIPE, PLIN1*
16	*NR0B2, SERPINE1*	
17	*CXCL8, LIPE, PARP2*	
18	*ADIPOR2, ATP6V0A2, HLA-DPB1, RIMS2*	*SETX, TNF*
19	*CXCL8, DIO2, INSR, LIPE*	
20	*HDAC3, LIPE*	
21	*B4GALT7*	
22	*PIK3R1*	
23	*ADIPOR2, INSR, LMNB2*	
24	*ADIPOR2, CCL3, CXCL8, IL6*	*LEP*
25	*IL18*	
6	*ATP6V1A, PPARA, SPRTN*	
7	*INSR, LEPR, PIK3R1, PPARA*	*USP8*
8	*RIMS2*	
9	*CCL3, FBN1, LIPE*	
10	*ADIPOR2, DIO2, INSR, LIPE*	*LMNB2, LPL, SERPINE1, TYMS,*
11	*CCL3, FBN1, LIPE*	

**Figure 4.**
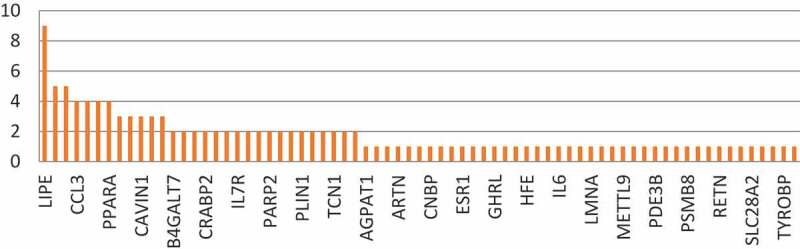
Frequency of lipodystrophy genes in diabetic patients showing ±2 fold expression difference from the average mean expression in the control group

These genes were further analysed for their frequencies in the diseases population. (Figure 4)

**Figure 5.**
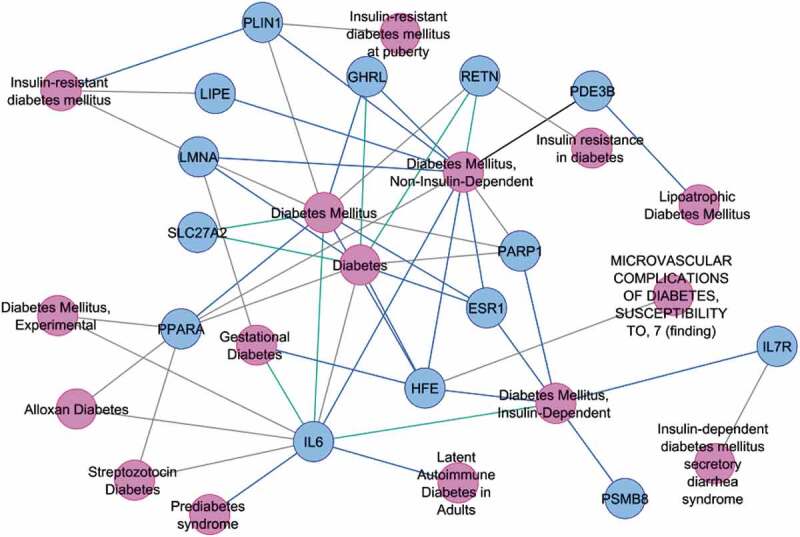
Associations of lipodystrophy genes (showing ±2 fold expression difference from the average mean expression in the control group) with Type 2 diabetes and its related phenotypes in diabetic patients

Interestingly 14 of these 24 lipodystrophy genes have been annotated to type 2 diabetes and its related phenotypes in DisGeNET, clearly supporting our hypothesis of common thread between both the diseases. (Figure 5)

## Discussion

The findings of this study can be summarized as follows: (1) Out of the total 138 lipodystrophy genes, seven and nine genes were respectively up and downregulated in the peripheral subcutaneous adipose tissue of T2D patients (2) There was approximately an 18% overlap between DEGs and the physical and genetic interactome of lipodystrophy genes, (3) On WGCNA analysis, several modules of lipodystrophy genes showed correlation with disease-related intermediate phenotypic traits among the diabetics, but not in the control group and (4) Individualization of T2D patients is possible on the basis of altered expression of one or more of these genes in the peripheral subcutaneous adipose tissue. Taken together, these findings suggest that not only there is a significant genetic overlap between partial lipodystrophy and the ‘thin fat phenotype’ of Asian Indian diabetics, but there are also indications for the role of altered peripheral subcutaneous adipose tissue expression of these lipodystrophy genes in the pathogenesis of T2D in this population.

Lipodystrophies are rare monogenic diseases caused by mutations in the coding sequence of certain genes, which were identified by linkage analysis and are pedigree specific [2,3]. These genetic defects are associated with a relatively severe metabolic disorder manifesting early in life. The genome to phenome correlation in most of the subjects suffering from this disease is well understood. The primary pathophysiological defect in these diseases is the inability of the subcutaneous adipose tissue to store excess calories as fat and the consequent ectopic fat deposition in skeletal muscle, liver, pancreas, etc. We recently hypothesized that lipodystrophy could be considered as a relevant model for studying molecular pathophysiology of Metabolic Syndrome [7]. The finding of this study that there exists an overlap of around 18% between the physical and genetic network of these genes and the differentially expressed genes (DEGs) in the subcutaneous adipose tissue of diabetics suggests that both these diseases are clinical manifestations of two different genetic defects in a common physiological process. In lipodystrophy, it is a mutation in the coding sequence of a single gene in this physiologic process while in diabetes, it is the altered expression of a group of these genes in the subcutaneous adipose tissue. We have shown that when lipodystrophy genes are subjected to GeneMANIA – a network prediction database, the lipodystrophy genes form a network with other genes and among themselves. All these genes are known to influence one or the other aspects of fat metabolism in adipose tissue. These genes and their activators were found to enrich the process of adipogenesis. On the other hand, we recently found that in the peripheral subcutaneous adipose tissue of diabetics, the co-expressed modules of differentially expressed genes not only show correlation with diabetes and its intermediate phenotypic traits, but a majority of them were found to enrich the process of adipogenesis and other aspects of fat storage in subcutaneous adipose tissue. Taken together all these findings suggest that both lipodystrophy and ‘thin fat phenotype’ of Asian Indian diabetics are genetic disorders of adipogenesis and fat storage in peripheral subcutaneous adipose tissue, the former is a monogenic coding sequence mutation and the latter is a polygenic functional defect. In other words, both can be clubbed together under the umbrella term ‘*Genetic disorders of Adipogenesis and fat storage*’.

Adiposopathy (or sick fat) is defined as adipocyte and adipose tissue anatomical and functional disturbance promoted by positive caloric balance in genetically and environmentally susceptible individuals [29]. The findings of the present study support the concept that ‘Asian Indian thin fat phenotype’ represents adiposopathy in a sense that primary pathophysiological defect in lipodystrophy is adipogenesis failure or the inability to store calorie excess as fat and the same mechanism is shared by T2D subjects also. The finding of this study; that the modules of co-expressed lipodystrophy genes showed association with intermediate phenotypic traits of diabetes in diabetics but not in the control group further supports the concept that adipose tissue dysfunction is one of the major pathophysiological determinants of diabetes in them. However, there are several limitations of the above interpretation of shared molecular mechanism between the lipodystrophy syndrome and the thin fat phenotype of Asian Indian diabetics. Only about 10% of lipodystrophy genes were differentially expressed in the thigh adipose tissue of diabetics, even the overlap between their physical and genetic network and DEGs was only 18%. One explanation is that the underlying genetic defect in the majority of patients suffering from lipodystrophy remains unknown. Studying genetic polymorphs of DEGs in thigh adipose tissue of diabetics or their regulatory regions could be a potential strategy to identify them. Another argument could be that some of the T2D subjects in our study might have been suffering from partial lipodystrophy syndrome. However, as discussed below, on individualization almost all the diabetics showed significantly altered expression of at least one lipodystrophy gene.

Another important finding of this study was – the individualization of diabetic patients based on 2 fold above or below the average of the control population for lipodystrophy genes. Interestingly, almost 83% of patients in this study could be individualized based on the altered expression of 1 to 19 lipodystrophy genes. This type of analysis is clinically relevant as it gives a ray of hope to individualize diabetics based on precise molecular pathology and pharmacotherapy directed for this specific defect can be instituted, *i.e*. thiazolidinediones for PPAR-γ defects. However, there are several limitations to this proposal. We could not measure the functional significance of the expression level of individual lipodystrophy gene with diabetes-related intermediate phenotypic traits, relatively small size of the study and lastly the use of relatively older technology of microarray. However, it lays the foundation of a new concept of individualization of diabetics based on altered expression of genes involved in adipogenesis and adipose tissue fat storage dysfunction.

In conclusion, we found that some of the lipodystrophy genes are differentially expressed in the peripheral subcutaneous adipose tissue of diabetics and there is a significant overlap between the physical and genetic interactome of the lipodystrophy genes and the DEGs in this tissue. Association of modules of co-expressed lipodystrophy genes with diabetes-related intermediate phenotypic traits suggests their role in the pathophysiology of diabetes. Individualization of diabetics based on the expression of a single or a few lipodystrophy genes is interesting and needs further investigations.

## Supplementary Material

Supplemental Material
